# Enhancing organizational capacity to provide cancer control programs among Latino churches: design and baseline findings of the CRUZA Study

**DOI:** 10.1186/s12913-015-0735-1

**Published:** 2015-04-09

**Authors:** Jennifer D Allen, Maria Idali Torres, Laura S Tom, Sarah Rustan, Bryan Leyva, Rosalyn Negron, Laura A Linnan, Lina Jandorf, Hosffman Ospino

**Affiliations:** Dana-Farber Cancer Institute, Boston, MA USA; Department of Public Health and Community Medicine, Tufts University, 112 Packard Ave, Medford, MA 02155 USA; Mauricio Gaston Institute for Latino Community Development and PublicPolicy, University of Massachusetts, Boston, MA USA; National Cancer Institute, Bethesda, MD USA; University of North Carolina at Chapel Hill, Chapel Hill, NC USA; Icahn School of Medicine at Mount Sinai, New York, NY USA; Boston College, Chestnut Hill, MA USA

**Keywords:** Latinos, Hispanics, Faith-based organizations, Catholic, Cancer screening, Evidence-based interventions, Implementation science, Organizational capacity enhancement, Capacity building, Community-based participatory research

## Abstract

**Background:**

Faith-based organizations (FBOs) have been successful in delivering health promotion programs for African Americans, though few studies have been conducted among Latinos. Even fewer have focused on organizational change, which is required to sustain community-based initiatives. We hypothesized that FBOs serving Latinos would be more likely to offer evidence-based strategies (EBS) for cancer control after receiving a capacity enhancement intervention to implement health programs, and designed the CRUZA trial to test this hypothesis. This paper describes the CRUZA design and baseline findings.

**Methods:**

We identified Catholic parishes in Massachusetts that provided Spanish-language mass (n = 65). A baseline survey assessed organizational characteristics relevant to adoption of health programs, including readiness for adoption, “fit” between innovation and organizational mission, implementation climate, and organizational culture. In the next study phase, parishes that completed the baseline assessment will be recruited to a randomized cluster trial, with the parish as the unit of analysis. Both groups will receive a Program Manual and Toolkit. Capacity Enhancement parishes will also be offered technical support, assistance forming health committees and building inter-institutional partnerships, and skills-based training.

**Results:**

Of the 49 parishes surveyed at baseline (75%), one-third (33%) reported having provided at least one health program in the prior year. However, only two program offerings were cancer-specific. Nearly one-fifth (18%) had an active health ministry. There was a high level of organizational readiness to adopt cancer control programs, high congruence between parish missions and CRUZA objectives, moderately conducive implementation climates, and organizational cultures supportive of CRUZA programming. Having an existing health ministry was significantly associated with having offered health programs within the past year. Relationships between health program offerings and other organizational characteristics were not statistically significant.

**Conclusions:**

Findings suggest that many parishes do not offer cancer control programs, yet many may be ready to do so. However, the perceptions about existing organizational practices and policies may not be conducive to program initiation. A capacity enhancement intervention may hold promise as a means of increasing health programming. The efficacy of such an intervention will be tested in phase two of this study.

## Background

Evidence-based strategies (EBS) to increase utilization of cancer screening tests have proliferated rapidly over the past 20 years [1]. However, the utility of these strategies is hindered by limited information about how best to deliver and sustain them within existing community infrastructures, where they could exert maximum benefit. Historically, there has been greater emphasis on developing and testing new interventions under “ideal” (controlled) conditions, with little focus on *if* or *how* interventions could be transferred or scaled for “real world” settings. As a result, little is known about how to encourage and support community organizations in adopting and implementing EBS. It is imperative to conduct research to understand the processes by which community organizations decide to adopt and implement EBS, rather than continuing to create complex interventions that are not sustainable outside the context of research trials. If the science of dissemination and implementation continues to lag behind that of intervention development, the ultimate aims of reducing cancer morbidity and mortality cannot be fully realized.

Collaborations with faith-based organizations (FBOs) has been suggested as an effective means for reaching out to underserved populations that experience health disparities, such as Latinos [2]. Several factors make FBOs natural partners for the delivery of cancer control programs. First, national data show that many FBOs view health as integral to their mission and are interested in providing health programs for their members, yet only a small percentage actually do [3,4]. Second, FBOs often have infrastructures (e.g., health ministries), facilities (e.g., meeting halls), and social/human resources (e.g., support networks) that may be useful for delivering and sustaining health programs over time. Moreover, FBOs often place a high value on volunteerism— which can bolster personnel resources needed for program delivery [5,6] and potential sustainability of programs once grant funding is completed [2].

Despite the potential for establishing cancer control interventions through FBOs, minimal organizational-level research has been conducted on how best to harness this resource. A number of randomized trials of health interventions in African American churches have successfully targeted changes in individual health behaviors [7], such as diet and physical activity [3,4,8-17], as well as cancer screening [18-23]. However, few have targeted organizational-level change [24,25]. Moreover, only a handful of intervention trials have been conducted in Latino FBOs; of these, all have targeted individual-, not organizational-level change [26-33].

In response to the paucity of research on organizational interventions in Latino FBOs, we designed CRUZA, a three-year study of Catholic churches in Massachusetts. We targeted Catholic churches (hereafter referred to as “parishes”) since a little over half of all Latinos in the U.S. self-identify as Catholic [5,34,35]. The name “CRUZA” was selected because of its religious connotations (“la cruz” in Spanish means “cross” in English) but also to reflect the collaboration among FBOs, academic universities, and community organizations. CRUZA’s aims are to understand the organizational infrastructure, skills, and resources required by parishes to adopt and implement EBS for cancer control. The trial will compare the efficacy of a Capacity Enhancement intervention versus Standard Dissemination approach in promoting EBS adoption and implementation. In this paper, we describe the study rationale, design, methods and baseline findings, including a description of organizational characteristics of FBOs, as well as their past offering of health programs.

## Method

### Overview of design

**Figure 1:**
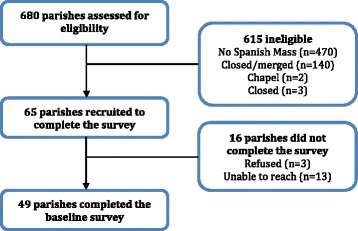
**Study schema, sampling and recruitment results, CRUZA Study.**

In the first phase of CRUZA, we identified Catholic parishes in Massachusetts offering religious services in Spanish and surveyed parish leaders to: (1) document the prevalence of health programming and EBS for cancer control; and (2) understand organizational characteristics associated with implementation of health programs. This manuscript focuses on findings from phase one. Phase two is a randomized cluster trial comparing a 3-month Capacity Enhancement (CE) Intervention with a Standard Dissemination (SD) comparison condition at the organizational level, with the parish as the unit of randomization and analysis. See Figure 1 for the parent study schema. The Institutional Review Board at the Harvard School of Public Health approved all study protocols (protocol number: 19674).

### Conceptual framework

CRUZA’s conceptual framework integrates theories of organizational change [36], diffusion of innovations [37], and the Consolidated Framework for Implementation Research (CFIR) [3]. The CFIR is a meta-theoretical implementation framework that integrates constructs from relevant theories and addresses multiple domains, including the: (1) characteristics of the intervention; (2) inner organizational setting; (3) outer setting; (4) characteristics of those implementing the intervention; and (5) processes put in place for implementation [38]. Taken together, these theories and frameworks suggest that interventions are more likely to be adopted and implemented if they are simple, adaptable, low-cost, easy to use, and demonstrate a relative advantage over current practices [37]. Moreover, they suggest that an organization that is stable, and has clear lines of decision-making and communication, capacity for and receptivity to change is more likely to adopt/implement an innovation than those lacking these characteristics. The outer setting – including connectedness with other organizations – may also influence implementation. Characteristics of individuals or groups charged with implementation of change (e.g., skills, self-efficacy, commitment) may also play a role. Processes for implementation refer to activities essential to complete intervention activities, including: planning, engaging, executing, reflecting and evaluating [38].

While the CFIR provides a model for examining factors that may influence the adoption and implementation process, the Reach Effectiveness Adoption Implementation Maintenance (RE-AIM) model [39] augments this by providing a framework for evaluating intervention uptake at the organizational level [40,41]. Relevant aspects of this framework include: (1) adoption – the absolute number, proportion, and representativeness of organizational units willing to initiate a program; (2) implementation – the delivery of the intervention as planned; and (3) maintenance – whether an intervention sustains over time and integrates into organizational practices [39]. Adoption, implementation, and maintenance are outcomes for the larger CRUZA trial.

### Community engagement

We employed a community-based participatory (CBPR) approach, in recognition of the need for community expertise and engagement in all phases of the study [42]. In the first year of the study, we established a statewide Community Advisory Board (CAB) comprised of Catholic faith leaders and representatives from health and social service organizations serving Latinos. CAB members met regularly to advise about recruitment strategies and materials, data collection methods, and religious tailoring of messages. Our community activities also included extensive formative research. Between April 2011–February 2012, we conducted 18 key informant interviews among Latino faith and community leaders, as well as 8 focus groups totaling 67 Spanish-speaking parishioners [43]. These formative research activities illuminated contextually appropriate strategies for engaging faith communities and adapting EBS for delivery in parishes. These findings are described in detail elsewhere [43].

### Organizational recruitment

Study sites were located in Massachusetts, which has 577 Catholic parishes under the purview of four dioceses [44]. Given the hierarchical structure of the Catholic Church, we conducted diocesan (administrative units of parishes) recruitment prior to individual parish recruitment. During this period, investigators gave formal presentations at a state-wide gathering of more than 30 Catholic leaders. Investigators subsequently met individually with the bishops and/or Hispanic Ministry directors within each of the four dioceses in Massachusetts to obtain their approval and support.

Once permission for conducting the study in parishes was granted at the diocesan level, we utilized a two-stage sampling scheme to identify eligible parishes, and within them, eligible representatives to respond to the organizational surveys. This process is described in detail elsewhere [45]. First, using lists reviewed by the four dioceses, we identified 70 parishes that potentially met our eligibility criteria. They were: (1) Roman Catholic; (2) located in Massachusetts; (3) offered mass in Spanish; and (4) not undergoing closure/merger at the time of recruitment.

We mailed recruitment packets that included a project brochure, which described the study‘s goals and procedures and provided informed consent information. The packet also contained a letter of support from the bishop. We enclosed a request for contact information so that pastors could indicate if they were willing to participate, and if so, the names of appropriate parish representative(s) to complete relevant sections of the organizational survey.

Bilingual survey assistants subsequently made telephone calls and in-person visits to parishes, following study recruitment protocols and using standardized scripts. During these interactions, survey assistants described the study verbally and provided written recruitment materials. If the pastor consented to proceed with study activities, we asked for the names of organizational representatives who could accurately describe parish activities and organizational characteristics for the organizational surveys.

### Data collection

We contacted the organizational representatives named by the pastor by phone or in person using a standardized script. Detailed tracking logs ensured that contact attempts occurred at various times of the day and week. During calls/visits, we presented study information, ascertained level of interest, and offered to conduct the interview immediately or schedule at another time convenient for the participant. Prior to interview administration, survey assistants obtained verbal informed consent in the respondent‘s preferred language (English/Spanish).

The organizational survey consisted of four sections based on the content and intended respondent: Part A – leadership (pastor), Part B – bookkeeping (business manager), Part C – Hispanic ministry (deacon/director of Hispanic ministry), and Part D – health/social services (parish nurse or director of social outreach). In cases where one individual completed all survey sections, the total interview time was approximately 60 minutes. When completed by different individuals, each segment took approximately 20 minutes. We donated a $50 gift card to the parish upon completion of any survey sections. A detailed description of the survey recruitment methods is available elsewhere [45].

For non-participating parishes, we collected information on finances, weekly attendance (when available), founding year of parish, presence of a health ministry/programs, and number of Spanish services from publicly available sources including parish newsletters, websites, and information documented in Diocesan reports.

### Measures

#### Instrument development

We reviewed the literature for validated instruments that assessed organizational characteristics associated with adoption and implementation of innovations among community organizations. We also identified existing national surveys conducted in FBOs and catalogued measures on health-related activities and characteristics of churches [46,47]. Available instruments were reviewed by the investigator team, the CAB, and our Scientific Advisory Committee – experts in the fields of organizational behavior, cancer control and faith-based interventions. Following an iterative review process, we conducted cognitive testing of the resulting survey with experienced pastoral leaders outside our sample (N = 5) to assess item comprehension and cultural/religious appropriateness of items. Afterward, items were refined and subsequently re-tested with two parish leaders (also not included in our sample). The entire instrument was reviewed by the CAB and the Scientific Advisory Committee, with particular attention to the relevance and “face validity” of the Organizational Characteristics measures. Final data collection instruments were vetted by officials from the Archdiocese of Boston and translated into Spanish by a certified translator working for the Archdiocese.

#### Organizational characteristics

We assessed organizational characteristics that emanated from our conceptual model, including: (1) *organizational readiness* – 12 items measuring the organizational members’ shared resolve to implement a change (change commitment) and collective capability to do so (shared efficacy), adapted from Weiner et al. [48,49]; (2) *innovation-values fit* – 5 items based on the work of Belkhodja et al. measuring the extent to which health programs fit with the overall organizational mission and values [50]; (3) *implementation climate* – 7 items adapted from Weiner et al. measuring the extent to which the organization‘s policies and practices encourage, support, and reward implementation of programs [51]; and (4) *organizational culture* – 7 items adapted from the work of Helfrich et al. measuring the extent to which the organization has an atmosphere of trust, support, flexibility, participative decision-making, and innovation, and that proper values are in place to optimize implementation [52]. Each item was read to respondents in the form of a statement and respondents were asked about the extent to which they agreed with the statement on a five-point Likert scale (1 = low agreement, 5 = high agreement).

For each construct, items were summed and divided by the total number of items in the scale, with 1 representing the lowest level of a given factor and 5 representing the highest. For each of the Organizational Characteristics, responses (ranging from 1–5) were summed and subsequently divided by the total number of items in the scale. Therefore, the theoretical range for each of the Organizational Characteristics was 1–5.

We also measured parish resources (e.g., size, monetary collections, volunteerism), leader characteristics (e.g., educational level, number of pastoral staff); existing health-related structures or committees (e.g., health ministry), and existing/prior inter-organizational ties and collaborations with hospitals or health centers. Questions were worded so that it was clear that we were asking about the parish *organization* (i.e., organizational-level data), as opposed to individual opinions about these topics.

### Intervention development

EBS for cancer control adapted and packaged for use among CRUZA parishes were largely based on recommendations by the Community Preventive Services Task Force for breast, cervical, and/or colorectal cancer [53]. These included: (1) group education (although evidence insufficient for colorectal cancer); (2) one-to-one outreach; (3) small media; (4) client reminders; and (5) reduction of structural barriers (see Table 1). We conducted a systematic search for all research-tested intervention protocols for these EBS by scouring the online archives of Research Tested Intervention Programs (RTIPS) [54] and Cancer Control P.L.A.N.E.T. (Plan, Link, Act, Network with Evidence-based Tools) [55], and by contacting prominent intervention researchers in the field [56,57]. Through this process, we identified 28 community interventions for cancer control, which we then evaluated for cultural and linguistic appropriateness for Latinos and for delivery in a faith-based setting.

**Table 1:** **Content of CRUZA Toolkit**

**Evidence-based strategies** ^*^		**Contents of toolkit**
Small media	Videos and printed materials such as letters, brochures, and newsletters	•Bookmarks
		•Parish bulletin inserts
		•Brochures/tip sheets
		•Posters
		•Videos
		•Magnets
Group education**	Presentations, lectures and other interactive formats conducted by health professionals or trained laypeople	•Listing of available guest speakers
		•Videos
		•Cancer knowledge bingo game
Reminders	Written or telephone messages advising people that they are due for screening	•Birthday bulletin inserts
		•Birthday cards
		•Reminders from pastor delivered from the pulpit
One-on-one education	Delivery of information by health professionals, lay health advisors, or volunteers by telephone or in person in medical or community settings	•Scripts and FAQs for conversations after Mass
Reducing structural barriers	Facilitating access by addressing non-economic burdens that make it difficult for people to access cancer screening (e.g., distance, time, language)	•Strategies for building partnerships with local health center, service organizations, interpreters
		•Strategies for recruiting volunteers to assist with transportation & childcare
		•Planning guide for conducting community health fair
		•Contact information for cancer screening vans
		•Liaisons who enroll individuals in health insurance

*Adapted from: Guide to Community Preventive Services. Cancer prevention and control: client-oriented interventions to increase breast, cervical, and colorectal cancer screening. www.thecommunityguide.org/cancer/screening/client-oriented/index.html.

**Although the Guide to Community Preventive Services acknowledges that there is insufficient evidence to support the efficacy of group education for colorectal cancer screening, we have included these activities as they been found efficacious among Latino populations.

We found that none of the standalone interventions met our criteria of: (1) having been developed specifically for use among Spanish-speaking Latinos; *and* (2) having been tested in faith-based settings. We thus systematically adapted the EBSs following NCI’s “Using What Works” [58] guidelines to ensure suitability for a Spanish-speaking Latino audience in a Catholic parish setting. This adaptation process maintained core elements (those integral to the internal logic of the intervention) while modifying adaptive intervention elements (non-essential features that can be tailored without diluting the intervention‘s impact) [59].

We packaged EBS materials in a user-friendly CRUZA Program Manual and ToolKit, written at the 6^th^ grade level in both English and Spanish. The Program Manual provides a step-by-step activity guide for each EBS, and includes planning tools, sample materials, and resource guides. The CRUZA Toolkit is stocked with promotional and intervention materials that can be easily distributed to parishioners, such as: Bible bookmarks, parish bulletin inserts, spiritually-themed photo frames with health messages, birthday cards with reminders about age-appropriate screening guidelines, and bi-fold brochures that weaved family, faith, and health messages together. Toolkits contain local resource guides, including contact information for organizations willing to provide guest speakers, assistance with obtaining health insurance, and sources for additional health information and materials. Materials developed or translated by CRUZA are included on USB flash drives so parish leaders can tailor materials or print additional copies. Materials from MIYO are also included in the kit. Developed by health communications researchers at Washington University in St. Louis, MIYO enables individuals to customize small media and client reminders using an intuitive user interface and an extensive library of audience-tested images, messages and designs [57]. Table 1 summarizes the EBS and how they are packaged in the Toolkit. All materials have been translated by certified Spanish translators and reviewed by investigators (MIT, HO, RN) for accuracy, literacy level, and cultural and religious appropriateness.

### Treatment conditions

#### Capacity enhancement intervention

Parishes assigned to the Capacity Enhancement (CE) condition will receive the CRUZA Program Manual and Toolkit, as well as a standardized menu of CE activities available from a CRUZA Intervention Specialist. Designed to enhance parish capacity to deliver cancer control EBSs and based on “best practices” reported in the literature, [24] the menu includes: (1) technical assistance; (2) assistance with forming a health committee or ministry; (3) facilitation of inter-institutional partnerships; and (4) skill-building workshops. Parishes in the CE condition will *not be required* to access any of the menu’s activities. Rather, they will be able to select and choose any or all of the activities on the menu.

#### Technical assistance (TA)

CRUZA Intervention Specialists will provide individual guidance to parish liaisons with the goal of imparting knowledge and skills to implement CRUZA EBS. TA will be tailored to a parish liaison’s skillset, interests, and communication preferences. At a minimum, Intervention Specialists will offer monthly TA via telephone, in-person visits, or email.

#### Health committees

Health ministries are bodies within the parish that plan and execute health-related activities as part of the parish‘s overall mission [60]. In parishes with pre-existing health ministries, we will offer activities to expand their capacity to offer EBS. When forming a new health committee or ministry, we will ask pastors to recommend potential committee members, assist with recruiting these individuals, and plan/facilitate meetings.

#### Inter-institutional partnerships

We will encourage partnerships between parishes and existing community resources, such as local and state health departments, community health centers, local hospitals, and social service agencies. These organizations often have mutually beneficial goals, with community and health organizations seeking to reach underserved audiences, and parishes seeking ways to meet parishioners’ needs. These partnerships can assist individuals with enrolling in health insurance (mandatory in MA), find resources to reduce barriers to screening (e.g., transportation, mobile screening vans), and provide guest speakers to facilitate discussion groups. The CRUZA Intervention Specialist will help to identify local community partners and facilitate connections between the parish liaisons and local community groups.

#### Skill-building workshops

CRUZA Intervention Specialists will lead regional “Faith and Health” half-day workshops. These will target parish leaders and CRUZA parish liaisons. The purpose is to enhance organizational capacity for implementing and maintaining EBS for cancer control. Workshops will be guided by principles of empowerment [61,62] and adult learning [63]. The content will include: Catholic teachings on health and social justice, Latino health disparities, spirituality, and program planning.

#### Standard dissemination condition

Parishes assigned to the Standard Dissemination (SD) condition will receive a CRUZA Program Manual and Toolkit. In addition, they will be offered an initial consultation with a CRUZA Intervention Specialist. Subsequent requests for programmatic assistance during the intervention period will be referred to local community resources (e.g., American Cancer Society, community health centers, etc.).

### Process tracking

We developed a process tracking system to document: (1) number of health-related activities implemented during the intervention period (cancer control EBS, as well as other non-cancer control health activities); (2) the number of parishioners reached by health-related activities; and (3) fidelity to intervention protocols (as outlined in Program Manuals). These data will be used to understand implementation processes and can inform interpretation of the trial outcomes.

### Analysis

For this paper, we present data from the baseline organizational surveys. Using bivariate linear and logistic regression, we describe organizational characteristics from our conceptual model (latent constructs), other organizational characteristics (e.g., size, pastor’s level of education, etc.), and health program offerings within the past year among this sample of FBOs.

## Results

### Baseline organizational survey response rates

Interviewer-administered Organizational Surveys were completed for 49 out of 65 (75%) eligible parishes in phase one. Only 3 of the eligible parishes refused participation (4.6%). We were unable to confirm participation or refusal among pastors from an additional 13 parishes (20%) and as a result, cannot comment on their willingness to participate or reasons for refusal in the baseline organizational survey. Hence, using conservative assumptions, we count these 13 parishes among the ‘refusals’. Several individuals declined to respond to certain survey components; one refused to respond to the Bookkeeping section (component C) regarding church financial matters, and another declined to complete a section on Leadership Characteristics (component A), citing concerns regarding ability to complete it accurately. A detailed description of the parish recruitment and survey completion rates for the baseline organizational survey are published elsewhere [45].

### Baseline organizational characteristics

There was wide variation among participating parishes in several key characteristics (see Table 2). Parish size ranged from very small to very large (60 to 7741 people) and parishes ran the gamut of having very small Latino communities to being entirely Latino congregations (3%- 100%). Over three-quarters of the parishes reported having had a Hispanic ministry for ten years or more (76%), and the length of time that Spanish Masses have been offered ranged from 2 to 62 years. On average, parishes had 5.7 full-time paid pastoral staff. Pastors tended to be highly educated: 94% had a bachelor’s degree and nearly three-quarters had a graduate degree (74%). There were no significant differences between participating and non-participating parishes on any of the aforementioned characteristics.

**Table 2:** **Structural characteristics of participating parishes, CRUZA study, baseline survey (N = 49)**

	**Participating parishes**	
	**(N = 49)**	
**Resources**	**Mean**	***SD*** **(Range)**
Size of congregation	2020	1828 (60–7741)
Percent Latino	46	30 (1–100)
Percent of congregation that volunteer	9	15 (0–70)
Amount of weekly collection	5115	4037 (350–20000)
Years of Spanish Mass offered	29	14 (2–62)
**Parish leadership**		
Number of full-time paid pastoral staff	6	8 (0–44)
**Parish leaders**	%	
Percent of pastors with a graduate degree	73	
**Parish health programming**		
Percent of parishes with a health ministry	18	
Percent of parishes with health programs	33	
**Existing collaborations**		
Percent of parishes with hospitals or health center collaborations	69	

### Baseline health programming

Sixteen of the parishes (33%) reported having provided at least one health program in the past year. Among parishes that offered health programs, the mean number of programs was 1.9. Health education programs were those that focused on information about health topics (e.g., workshops), while health service programs were focused on the direct provision of health services (e.g., flu shots). The “other” category included programs with a health focus that did not fit into either of the other categories, such as nursing home visits or blood drives. The majority of programs were categorized as health education (n = 13; 42%) or health services (n = 11; 35%). Two of the health education programs were cancer-specific. In addition, 18% of the parishes reported having a health ministry that had been active in the previous year. See Table 3.

**Table 3:** **Number and types of health programs offered by participating parishes, CRUZA study, baseline survey (N = 49)**

	**Participating parishes**	
**Type of health program**	**(N = 49)**	
	***N***	**%**
Health Education	13	42%
Health Services	11	35%
Other*	7	23%
**Total health programs**	31	

*Examples of “Other” health programs include: blood drives, food pantries, nursing home visits, blood marrow donation, fundraising walks/events.

### Organizational variables and pastor characteristics associated with offering health programs

Overall, organizational readiness among all of the parishes was moderately high (mean = 3.7, *SD* = 0.97). Perceptions about the ‘fit’ between offering cancer control activities and parish mission was very high (mean = 4.4, *SD* = 0.76). Of all of the organizational variables hypothesized to be associated with EBS adoption and implementation, ratings of the implementation climate were rated lowest (mean = 2.8, *SD =* 1.20). When individual items of the implementation climate scale were examined, the two items that received the lowest scores addressed whether parishes received recognition for offering health activities (mean = 2.0, *SD =* 1.48) and whether financial support was available for these activities (mean = 2.3, *SD* = 1.77). More than half of the respondents disagreed entirely with the statements “My parish receives recognition” (59%) and “My parish gets the financial support they need” (59%) for health activities. The Cronbach’s alpha coefficient for each of the four latent organizational constructs was high, indicating a high internal consistency within each of the scales (see Table 4).

**Table 4:** Organizational characteristics*, participating parishes, CRUZA study, baseline survey (N = 49)

	**Participating parishes**		
**Organizational characteristics**	**(N = 49)**		
	**Mean**	**SD**	***Cronbach’s α***
Organizational readiness	3.72	0.97	0.96
Innovation and values fit	4.41	0.76	0.86
Implementation climate	2.83	1.20	0.82
Organizational culture	4.40	0.73	0.78

*Response categories: 1 = Low through 5 = High.

According to the results of linear regression analyses, only the presence of an existing health ministry was significantly associated with the number of health programs offered (*b* =0 .354, *t*(1) = 0.26, *p* = 0.025). The presence of an existing ministry explained a small proportion of the variance in the number of health programs offered by the parishes (*r2* = 0.106). Analyses to examine relationships between other organizational characteristics and health programming yielded no statistically significant relationships (data not shown).

### Structural characteristics of FBOs and associations with organizational constructs from conceptual model

We examined relationships between structural characteristics of parishes (e.g., size, percent Latino, etc.) with the latent organizational characteristics from our conceptual model. Results are presented in Table 5. Parishes with congregations comprised of more one-third Latino (<33%) reported significantly higher mean scores on Organizational Readiness compared with parishes that had fewer Latino members (mean = 4.05 versus 3.42, respectively; p < 0.05). Parishes with a health ministry reported slightly higher Organizational Readiness scores (mean = 4.23 versus 3.57), although this difference did not reach statistical significance (p < 0.10). Larger parishes and those with a greater proportion of Latino members were also marginally more likely to report Organizational Cultures conducive to offering EBS for cancer control (larger parishes mean = 4.52 versus 4.11; p < 0.10; more than a third Latino members mean = 4.47 versus 3.98; p < 0.10).

**Table 5:** **Associations between structural and organizational characteristics of parishes, baseline CRUZA study (N = 49)**

	**Organizational readiness**	**Innovation and values fit**	**Implementation climate**	**Organizational culture**	
Congregation size	Small	3.62 (19)	4.46 (20)	3.01 (20)	4.11 (20)~
	Large	3.71 (22)	4.24 (21)	2.65 (22)	4.52 (20)
Percent Latino	<33%	3.42 (14)*	4.34 (14)	2.78 (14)	4.37 (14)
	≥33%	4.05 (22)	4.43 (23)	3.04 (23)	4.46 (22)
Health ministry	Yes	4.23 (9)~	4.78 (9)	3.25 (9)	3.98 (9)~
Present	No	3.57 (35)	4.33 (35)	2.81 (36)	4.47 (34)
Health program(s)	Yes	3.79 (19)	4.41 (19)	2.83 (19)	4.20 (19)
Present	No	3.68 (29)	4.41 (29)	2.83 (29)	4.53 (28)
Weekly collections	< $5000	3.78 (24)	4.38 (25)	2.90 (25)	4.23 (24)
(English mass)	≥ $5000	3.66 (17)	4.36 (16)	2.75 (17)	4.47 (16)
Weekly collections	< $784	3.65 (32)	4.46 (32)	2.84 (32)	4.43 (31)
(Spanish mass)	≥ $784	3.83 (15)	4.33 (15)	2.91 (15)	4.30 (15)
Full-Time paid	Yes	3.71 (47)	4.40 (47)	2.83 (48)	4.38 (46)
Parish leader	No	3.83 (1)	4.80 (1)	-	5.00 (1)
Graduate degree,	Yes	3.72 (36)	4.36 (35)	2.71 (36)	4.35 (34)
Parish leadership	No	3.88 (9)	4.60 (10)	3.13 (10)	4.41 (10)
Existing	Yes	3.70 (10)	4.20 (10)	3.02 (10)	1.07 (10)
Collaborations	No	3.73 (38)	4.47 (38)	2.78 (38)	4.48 (37)

*p < 0.05.

~p < 0.10.

## Discussion

Evidence-based interventions must be translated from research settings into real-world community settings to have the greatest impact on human health. This first phase of the CRUZA study provides descriptive information about health programming offered by Catholic parishes, as well as an assessment of organizational characteristics theorized to be relevant to adoption and implementation of EBS for cancer control. These data will inform our efforts to develop, implement, and evaluate the efficacy of a capacity enhancement intervention as a means of promoting EBS uptake by parishes. To our knowledge, CRUZA is the first randomized trial of an organizational-level intervention aimed at disseminating EBS for cancer control in Latino Catholic communities.

Sixty-nine percent of eligible parishes invited to participate in the baseline assessment completed all four sections of the survey and 75% completed at least one survey section. These results suggest a willingness among Catholic parishes to take part in research and may portend interest in offering additional health programs. Indeed, one-third (33%) of parishes surveyed had conducted some form of health program within the past year. Although definitions of health programs differ somewhat across studies, our findings suggest a high level of commitment to health, when compared to a national study of a variety of denominations, which found that only 10% offered health programs [47]. Moreover, nearly one-fifth (18%) of parishes in this study had existing health ministries, which could be instrumental in the adoption, implementation, and maintenance of cancer control or other health programs over time. Overall, the results of the baseline surveys suggest that parishes willing to initiate EBS for cancer control, felt that the objectives of such programs aligned well with their organizational missions, and believed that their parish’s organizational culture was conducive to offering these programs. However, concerns about the feasibility of implementing programs given limited funds were prevalent and cannot be overlooked.

National studies have found that higher levels of education among FBO leaders and a greater proportion of volunteers relative to the size of the congregation are associated with health program offerings [11,47]. As we had expected given the rich history of social service provision in the Catholic Church, the proportion of volunteers relative to parish size was large in this sample, compared to churches nationally. Whereas half of congregations in the U.S. do not have volunteers, [47] the mean proportion of volunteers relative to the size of the congregation was 9% in this study. In addition, compared to the National Congregations Study, where 75% of religious leaders had a bachelors degree, [47] pastors in this study were highly educated, with 74% having a graduate level degree. This may well reflect the level of training that is required of Catholic pastoral staff and leaders. Notably, the vast majority of pastors completed the organizational surveys in English.

Parishes had high levels of existing collaborations with health/social service organizations, although these collaborations largely reflected the offering of communion, shared prayer and social support offered Catholic in institutional settings, such as hospitals, nursing homes, prisons etc., as opposed to health promotion activities. Relationships between FBOs and health or social service organizations in our sample may be due to affiliations between the Catholic Church and hospitals. The combination of favorable organizational characteristics, existing health ministries, high levels of education among pastors, and existing collaborations with health and social service agencies provides strong support for the premise that Catholic parishes may be effective partners in disseminating EBS for cancer control among Latinos. The finding that parishes with a greater proportion of Latino members reported higher Organizational Readiness to adopt EBS for cancer control also provides support for this hypothesis.

Before we discuss study implications, limitations must be noted. First, with only 49 parishes, we lacked adequate statistical power to detect small differences between health program offerings and organizational characteristics. In our calculations, our power to detect a medium effect size (0.40) was less than 40%. Nevertheless, the second phase of the study is adequately powered to detect meaningful changes in the primary outcomes of the trial (adoption and implementation of EBS). Second, in nearly one-third of the parishes (31%) that completed the organizational survey, one individual (most often the pastor) completed the entire assessment. When examining organizational-level factors, it is ideal to have multiple respondents to ensure that the data are not skewed toward the perceptions of an individual [64]. Another limitation is that these data were collected only among Catholic parishes. While the majority of Latinos living in the U.S. self-identify as Catholic, a growing sector of Latinos identify with non-Catholic Christian denominations whose structures, resources, and practices are different from those in the Catholic Church [5]. Additional research is needed to understand adoption and implementation of EBS beyond Catholic parishes.

Despite these limitations, the first phase of this study provides important information about the existing resources and needs of Catholic parishes in Massachusetts, as we embark on efforts to disseminate EBS for cancer control. Furthermore, we now have evidence that it is feasible to recruit parishes to participate in research activities. A detailed process tracking system is in place to evaluate intervention reach and dose in phase two, as well as fidelity to intervention protocol. This system will be essential for identifying factors that facilitate or impede implementation, as well as for explaining any unexpected effects of the intervention.

## Conclusion

Baseline findings suggest that many parishes do not currently offer cancer control programs, yet many demonstrate readiness to do so. A capacity enhancement intervention holds promise as a means to increase health programming. We developed the CRUZA Capacity Enhancement intervention based on published ‘best-practices’ for working with faith-based organizations [65]. The general literature suggests that building community capacity to offer health programs should entail technical assistance, formal skills training, and assistance forming inter-institutional partnerships [65-70]. Yet, to our knowledge, these strategies have not been formally tested among FBOs. After doing extensive formative research, we concluded that the success of CRUZA would depend largely on the appropriate adjustment of the interventions to core Catholic religious beliefs and values. This strategy allowed us to build on strengths and convictions existing in the communities to advocate for health, while avoiding theological and ethical tensions*.* Taking the time and effort to ensure that interventions are not only culturally relevant, but also relevant and meaningful from a religious and spiritual perspective, we believe, will ultimately enhance the acceptability, adoption and maintenance of CRUZA programs*.* Overall, findings from the first phase of CRUZA suggest that Catholic parishes could be strong partners in efforts to implement EBS for cancer control and other prevention programs among Latinos.

## Abbreviations

EBS: Evidence based strategies
FBOs: Faith-based organizations
RE-AIM: Reach effectiveness adoption implementation maintenance
CAB: Community advisory board
TA: Technical assistance
CE: Capacity enhancement
SD: Standard dissemination

## References

[1] Glasgow RE, Emmons KM (2007). How can we increase translation of research into practice? types of evidence needed. Annu Rev Public Health.

[2] Campbell MK, Hudson MA, Resnicow K, Blakeney N, Paxton A, Baskin M (2007). Church-based health promotion interventions: evidence and lessons learned. Annu Rev Public Health.

[3] Duru OK, Sarkisian CA, Leng M, Mangione CM (2010). Sisters in motion: a randomized controlled trial of a faith-based physical activity intervention. J Am Geriatr Soc.

[4] Wilcox S, Laken M, Parrott AW, Condrasky M, Saunders R, Addy CL (2010). The faith, activity, and nutrition (FAN) program: design of a participatory research intervention to increase physical activity and improve dietary habits in African American churches. Contemp Clin Trials.

[5] Changing Faiths: Latinos and the Transformation of American Religion [http://pewhispanic.org/reports/report.php?ReportID=75]

[6] Brown K, Adamczyk A (2009). Racial/ethnic differences in the provision of health-related programs among American religious congregations. J Sociol Soc Welfare.

[7] Campbell MK, Hudson MA, Resnicow K, Blakeney N, Paxton A, Baskin M (2007). Church-based health promotion interventions: evidence and lessons learned. Annu Rev Public Health.

[8] Whitt-Glover MC, Goldmon MV, Karanja N, Heil DP, Gizlice Z (2012). Learning and developing individual exercise skills (LADIES) for a better life: a physical activity intervention for black women. Contemp Clin Trials.

[9] DeHaven MJ, Ramos-Roman MA, Gimpel N, Carson J, DeLemos J, Pickens S (2012). The GoodNEWS (genes, nutrition, exercise, wellness, and spiritual growth) trial: a community based participatory research (CBPR) trial with African-American church congregations for reducing cardiovascular disease risk factors: recruitment, measurement and randomization (vol 32, pg 630, 2011). Contemp Clin Trials.

[10] Winett RA, Anderson ES, Wojcik JR, Winett SG, Bowden T (2007). Guide to health: nutrition and physical activity outcomes of a group-randomized trial of an internet-based intervention in churches. Ann Behav Med.

[11] Bopp M, Wilcox S, Laken M, Hooker SP, Saunders R, Parra-Medina D (2007). Using the RE-AIM framework to evaluate a physical activity intervention in churches. Prev Chronic Dis.

[12] Young DR, Stewart KJ (2006). A church-based physical activity intervention for African American women. Fam Community Health.

[13] Resnicow K, Jackson A, Blissett D, Wang T, McCarty F, Rahotep S (2005). Results of the healthy body healthy spirit trial. Health Psychol.

[14] Resnicow K, Jackson A, Wang T, De AK, McCarty F, Dudley WN (2001). A motivational interviewing intervention to increase fruit and vegetable intake through Black churches: Results of the eat for life trial. Am J Public Health.

[15] Resnicow K, Taylor R, Baskin M, McCarty F (2005). Results of go girls: a weight control program for overweight African-American adolescent females. Obes Res.

[16] Yanek LR, Becker DM, Moy TF, Gittelsohn J, Koffman DM (2001). Project Joy: faith based cardiovascular health promotion for African American women. Public Health Rep.

[17] McNabb W, Quinn M, Kerver J, Cook S, Karrison T (1997). The PATHWAYS church-based weight loss program for urban African-American women at risk for diabetes. Diabetes Care.

[18] Holt CL, Litaker MS, Scarinci IC, Debnam KJ, McDavid C, McNeal SF (2013). Spiritually based intervention to increase colorectal cancer screening among african americans: screening and theory-based outcomes from a randomized trial. Health Educ. Behav.

[19] Holt CL, Wynn TA, Litaker MS, Southward P, Jeames S, Schulz E (2009). A comparison of a spiritually based and non-spiritually based educational intervention for informed decision making for prostate cancer screening among church-attending African-American men. Urol Nurs.

[20] Campbell MK, James A, Hudson MA, Carr C, Jackson E, Oates V (2004). Improving multiple behaviors for colorectal cancer prevention among African American church members. Health Psychol.

[21] Derose KP, Fox SA, Reigadas E, Hawes-Dawson J (2000). Church-based telephone mammography counseling with peer counselors. J Health Commun.

[22] Campbell MK, Demark-Wahnefried W, Symons M, Kalsbeek WD, Dodds J, Cowan A (1999). Fruit and vegetable consumption and prevention of cancer: The Black Churches United for Better Health Project. Am J Public Health.

[23] Erwin DO, Spatz TS, Stotts RC, Hollenberg JA (1999). Increasing mammography practice by African American women. Cancer Pract.

[24] Allicock M, Campbell MK, Valle CG, Barlow JN, Carr C, Meier A (2010). Evaluating the implementation of peer counseling in a church-based dietary intervention for African Americans. Patient Educ Couns.

[25] Allicock M, Campbell MK, Valle CG, Carr C, Resnicow K, Gizlice Z (2012). Evaluating the dissemination of Body & Soul, an evidence-based fruit and vegetable intake intervention: challenges for dissemination and implementation research. J Nutr Educ Behav.

[26] Duan NH, Fox SA, Derose KP, Carson S (2000). Maintaining mammography adherence through telephone counseling in a church-based trial. Am J Public Health.

[27] Lopez VA, Castro FG (2006). Participation and program outcomes in a church-based cancer prevention program for Hispanic women. J Community Health.

[28] Sauaia A, Min SJ, Lack D, Apodaca C, Osuna D, Stowe A (2007). Church-based breast cancer screening education: impact of two approaches on Latinas enrolled in public and private health insurance plans. Prev Chronic Dis.

[29] Welsh AL, Sauaia A, Jacobellis J, Min SJ, Byers T (2005). The effect of two church-based interventions on breast cancer screening rates among Medicaid-insured Latinas. Prev Chronic Dis.

[30] Jandorf L, Ellison J, Shelton R, Thelemaque L, Castillo A, Mendez EI (2012). Esperanza y Vida: a culturally and linguistically customized breast and cervical education program for diverse Latinas at three different United States sites. J Health Commun.

[31] Davis DT, Bustamante A, Brown CP, Woldetsadik G, Savage EW, Cheng XG (1994). The urban church and cancer control - a source of social-influence in minority communities. Public Health Rep.

[32] Lujan J, Ostwald SK, Ortiz M (2007). Promotora diabetes intervention for Mexican Americans. Diabetes Educ.

[33] Daniels NA, Juarbe T, Moreno-John G, Perez-Stable EJ (2007). Effectiveness of adult vaccination programs in faith-based organizations. Ethn Dis.

[34] Taylor P, Lopez MH, Martínez JH, Velasco G (2012). When Labels Don‘t Fit: Hispanics and Their Views of Identity.

[35] Perl P, Greely JZ, Gray MM (2006). What proportion of adult hispanics Are catholic? a review of survey data and methodology. J Sci Study Relig.

[36] Emmons KM, Weiner B, Fernandez ME, Tu SP (2012). Systems antecedents for dissemination and implementation: a review and analysis of measures. Health Educ Behav.

[37] Rogers EM (2010). Diffusion of Innovations, 4th Edition.

[38] Damschroder LJ, Aron DC, Keith RE, Kirsh SR, Alexander JA, Lowery JC (2009). Fostering implementation of health services research findings into practice: a consolidated framework for advancing implementation science. Implement Sci.

[39] Glasgow RE, Vogt TM, Boles SM (1999). Evaluating the public health impact of health promotion interventions: the RE-AIM framework. Am J Public Health.

[40] Gaglio B, Shoup JA, Glasgow RE (2013). The RE-AIM framework: a systematic review of use over time. Am J Public Health.

[41] Glasgow RE, Klesges LM, Dzewaltowski DA, Estabrooks PA, Vogt TM (2006). Evaluating the impact of health promotion programs: using the RE-AIM framework to form summary measures for decision making involving complex issues. Health Educ Res.

[42] Minkler M, Wallerstein N (2010). Community-Based Participatory Research for Health: From Process to Outcomes.

[43] Allen JD, Leyva B, Torres MI, Ospino H, Tom L, Rustan S, et al: Religious beliefs and cancer screening behaviors among Catholic Latinos: Implications for faith-based interventions. Journal of health care for the poor and underserved In press. 10.1353/hpu.2014.0080 PMC4162660 24858865

[44] Kenedy PJ & Sons (2012). The Official Catholic Directory Anno Domini 2012.

[45] Allen JD, Rustan S, Tom LS, Leyva B, Galeas AV, Ospino H, et al: Recruiting and surveying Catholic parishes for cancer control initiatives: Lessons learned from the CRUZA Implementation Study. In press 10.1177/1524839915582174 PMC5370546 25878192

[46] Chaves M, Anderson SL (2008). Continuity and change in american congregations: introducing the second wave of the national congregations study. Sociol Relig.

[47] Trinitapoli J, Ellison CG, Boardman JD (2009). US religious congregations and the sponsorship of health-related programs. Soc Sci Med.

[48] Weiner BJ, Amick H, Lee S-YD (2008). Review: conceptualization and measurement of organizational readiness for change: a review of the literature in health services research and other fields. Med Care Res Rev.

[49] Weiner BJ (2009). A theory of organizational readiness for change. Implement Sci.

[50] Belkhodja O, Amara N, Landry R, Ouimet M (2007). The extent and organizational determinants of research utilization in Canadian health services organizations. Sci Commun.

[51] Weiner BJ, Belden CM, Bergmire DM, Johnston M (2011). The meaning and measurement of implementation climate. Implement Sci.

[52] Helfrich CD, Li YF, Mohr DC, Meterko M, Sales AE (2007). Assessing an organizational culture instrument based on the Competing Values Framework: Exploratory and confirmatory factor analyses. Implementation Science.

[53] The Guide to Community Preventative Services. Cancer Prevention and Control: Client-oriented interventions to increase breast, cervical, and colorectal cancer screening. [http://www.thecommunityguide.org/index.html]

[54] Research-tested Intervention Programs (RTIPs). [http://rtips.cancer.gov/rtips/index.do]

[55] Cancer Control P.L.A.N.E.T. [http://cancercontrolplanet.cancer.gov/index.html]

[56] McNeill LH, Coeling M, Puleo E, Suarez EG, Bennett GG, Emmons KM (2009). Colorectal cancer prevention for low-income, sociodemographically-diverse adults in public housing: baseline findings of a randomized controlled trial. BMC Public Health.

[57] “MIYO: Make It Your Own” ‖ [http://miyo.gwb.wustl.edu/]

[58] Using what works: Adapting evidence-based programs to fit your needs. [http://cancercontrol.cancer.gov/use_what_works/start.htm]

[59] McKleroy V, Galbraith JS, Cummings B (2006). Adapting evidence-based behavioral interventions for new settings and target populations. AIDS Educ Prev.

[60] Catanzaro AM, Meador KG, Koenig HG, Kuchibhatla M, Clipp EC (2007). Congregational health ministries: a national study of pastors‘ views. Public Health Nurs.

[61] Wallerstein N, Bernstein E (1988). Empowerment education: Freire‘s ideas adapted to health education. Health Educ Q.

[62] Freire P (1985). Pedagogy of the oppressed. [Pedagogia do oprimido.].

[63] Vella J (2002). Learning to Listen, Learning to Teach: The Power of Dialogue in Educating Adults.

[64] Edwards JE, Thomas MD, Rosenfeld P, Booth-Kewley S (1997). How to Conduct Organizational Surveys: A Step-by-Step Guide.

[65] Fink B, Branch AY. Promising Practices for Improving the Capacity of Faith- and Community-Based Organizations. Roanoke, VA: Branch Associates, Inc. Prepared for: Administration for Children and Families, US Department of Health and Human Services; 2005.

[66] Wandersman A, Duffy J, Flaspohler P, Noonan R, Lubell K, Stillman L (2008). Bridging the gap between prevention research and practice: the interactive systems framework for dissemination and implementation. Am J Community Psychol.

[67] Maclellan-Wright MF, Anderson D, Barber S, Smith N, Cantin B, Felix R (2007). The development of measures of community capacity for community-based funding programs in Canada. Health Promot Int.

[68] Vega MY (2009). The CHANGE approach to capacity-building assistance. AIDS Educ Prev.

[69] Ayala G, Chion M, Diaz RM, Heckert AL, Nuno M, del Pino HE (2007). Accion Mutua (Shared Action): a multipronged approach to delivering capacity-building assistance to agencies serving Latino communities in the United States. J Public Health Manag Pract.

[70] Capacity building for diabetes outreach: a comprehensive tool kit for organizations serving Asian and Pacific Islander communities. [http://www.cdc.gov/diabetes/ndep/CE_aapiToolkit.htm#10]

